# Gouty Tophus in the Small Bowel Mimicking a Calcifying Mesenteric Mass

**DOI:** 10.7759/cureus.82288

**Published:** 2025-04-15

**Authors:** Vanessa Diller, Daniel Perez, Alexander Harms, Luca M Tavernar, Sven Petersen

**Affiliations:** 1 General and Visceral Surgery, Asklepios Hospital Altona, Hamburg, DEU; 2 Pathology, MVZ HPH Institute for Pathology and Hematopathology, Hamburg, DEU

**Keywords:** chronic gout, gout crystal, gout disease, small bowel resection, surgery

## Abstract

Gout is a common metabolic disorder caused by hyperuricemia, which results in the deposition of monosodium urate crystals in various parts of the body. In the present case, a large gout tophus was detected based on gout disease. Although gouty tophi might occur in many locations during a long-standing gout disease, the vast majority are located in the articular tissue. An 80-year-old female patient was admitted to the emergency room complaining of persistent nausea and vomiting for two days. In addition, she reported abdominal pain and cramps that she had not experienced before. A CT scan revealed a large calcified mesenteric mass in the right side of the abdomen. The patient underwent surgery and removal of the mesenteric mass including 55-cm jejunal resection. Histologically, sections showed a capsule-like boundary of varying widths, mostly cell-poor lesions, as well as siderophages and strong lymphatic infiltrate at the margins, corresponding to residual lymph node tissue. In the periphery of the lesion, brace-shaped dystrophic calcifications completed the histological image of a gouty tophus. In this case report, we describe an extremely rare case of a mass in the area of the small intestine mesentery caused by monosodium urate crystals. Even if patients do not present with articular manifestation of gout, deposition in other tissues is possible and presents a risk factor for the development of diseases. For patients with unclear abdominal masses suffering from hyperuricemia, despite being rare, a gout tophus should be thought of.

## Introduction

Gout is a common metabolic disorder caused by hyperuricemia, which leads to the deposition of monosodium urate crystals in various tissues. This condition is particularly prevalent in developed countries, for instance, approximately 4% of adults in the United States are affected [1,2]. Gout tends to occur more frequently with increasing age [3]. In women, it typically manifests after menopause, while in men, incidence increases after the age of 30 [1,4].

Risk factors for developing gout include genetic predisposition, obesity, and comorbidities such as hypertension, diabetes, kidney disease, and metabolic syndrome [5,6]. The rising prevalence of gout in recent decades may be linked to the growing incidence of obesity and diabetes [7]. In addition to acute gout attacks, the disease often follows a chronic course. This chronic form typically involves recurrent inflammation of the same joints, which may eventually lead to joint damage and the formation of gouty tophi. These tophi primarily develop in articular structures, particularly in the small joints of the extremities, such as fingers and toes, causing inflammation and arthritis [8,9]. Non-articular structures, including the kidneys, bones, and cartilage, may also be affected by urate crystal deposition [10].

Common differential diagnoses include activated osteoarthritis (without elevated uric acid levels), classic rheumatoid arthritis, and infectious (bacterial) arthritis. Systemic diseases such as systemic lupus erythematosus or psoriatic arthritis can also mimic the clinical picture of gout [11].

Diagnosis involves both clinical and laboratory findings. While elevated serum uric acid levels can support the diagnosis, typical joint involvement patterns are also crucial [12]. However, it is important to note that uric acid levels may be normal or even low during acute flares. Further diagnostic tools include joint aspiration to detect urate crystals, as well as imaging techniques such as ultrasound or X-rays to assess joint damage.

Management depends on the disease phase. For chronic gout, urate-lowering therapy is essential to maintain low uric acid levels, prevent recurrent attacks, and minimize joint damage [13]. During acute episodes, anti-inflammatory medications, including nonsteroidal agents such as ibuprofen or naproxen and corticosteroids such as prednisone, are effective in relieving pain and inflammation [14]. Colchicine remains a second-line option for rapid symptom relief during acute flares.

Here, we report an unusual case involving a large mesenteric mass in the small bowel caused by urate crystal deposition.

## Case presentation

An 80-year-old female patient presented to the emergency room with persistent nausea and vomiting lasting for two days, accompanied by new-onset abdominal pain and cramps. She denied any significant changes in her bowel movements. On clinical examination, her vital signs were within normal limits, and she showed tenderness in the lower middle of the abdomen without signs of acute peritonitis. The patient’s medical history included hypertension, hypercholesterolemia, and hyperuricemia. She had previously undergone cholecystectomy, appendectomy, and laparotomy with adhesiolysis for small bowel obstruction two years prior. She had been on a daily 100 mg dose of allopurinol for over 10 years to manage hyperuricemia, and previous blood tests indicated moderately elevated uric acid levels.

**Figure 1:**
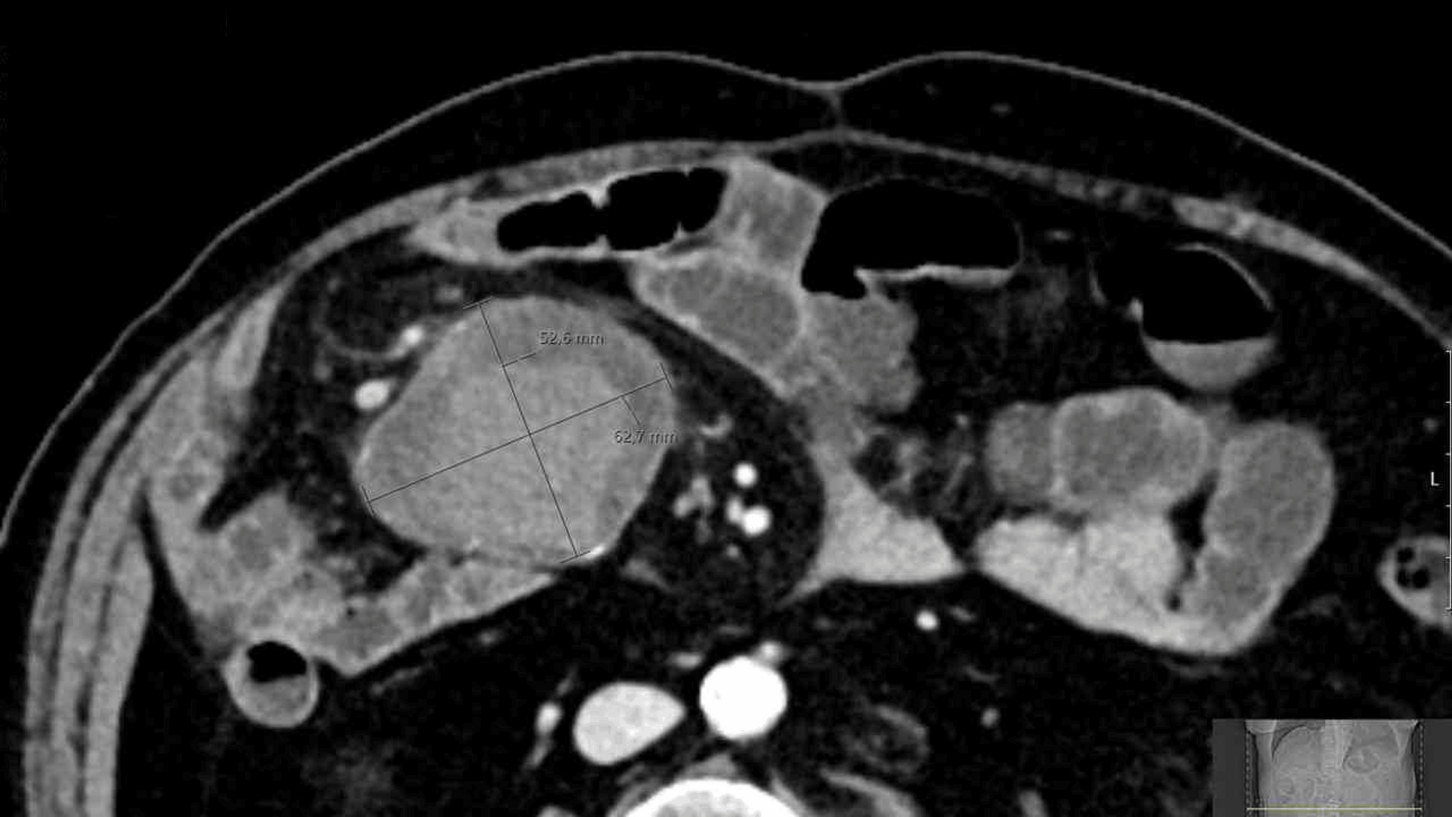
CT scan showing a solid, inhomogeneous mass in the right side of the mid-abdomen measuring 6.3 × 5.3 × 4.8 cm.

Initial lab results showed only a slightly elevated CRP level (18.4 mg/dL; reference <5 mg/dL), and the urate level was within normal limits (<5 mg/dL). Ultrasound examination revealed an unclear mass in the right side of the mid-abdomen. A CT scan with contrast further characterized the mass as a solid, inhomogeneous mass measuring 6.3 × 5.3 × 4.8 cm (Figure 1). The mass exhibited significant calcification and contrast enhancement, with a central Hounsfield unit value of 70 and a peripheral value of 30. Surrounding lymph nodes showed minimal enhancement. No organ or vascular invasion was observed.

Given the subacute presentation, the patient was scheduled for surgery. The differential diagnoses included mesenteric lymphoma, gastrointestinal stromal tumor (GIST), mesenteric sarcoma, or metastasis from an undiagnosed tumor.

Surgery

**Figure 2:**
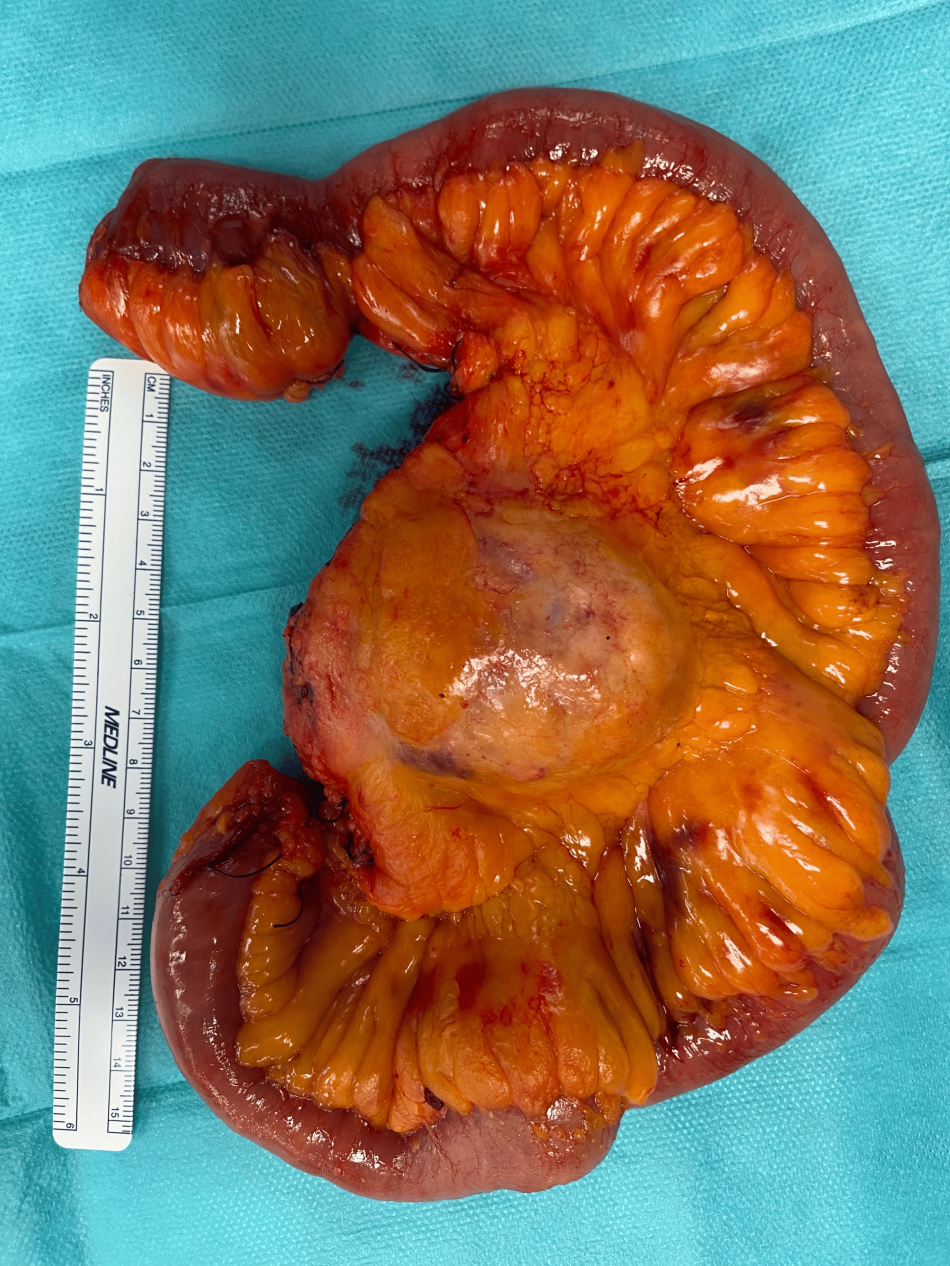
Specimen from small bowel resection showing a 7-cm mass in the jejunal mesentery.

The patient underwent laparotomy at the Department of General and Visceral Surgery, and the tumor was found in the mesenteric tissue of the jejunum, without involvement of the bowel wall. As seen on the CT scan, the mass was closely related to the middle colonic artery but did not infiltrate or encase it. The 7-cm mass was excised en bloc with a 50-cm segment of the jejunum (Figure 2), starting the resection approximately 70 cm below the duodenal-jejunal flexure. The mesenteric artery was preserved, and an end-to-end jejuno-jejunal anastomosis was performed to restore intestinal continuity. The postoperative course was uncomplicated, and the patient was discharged on the sixth postoperative day.

Histology

**Figure 3:**
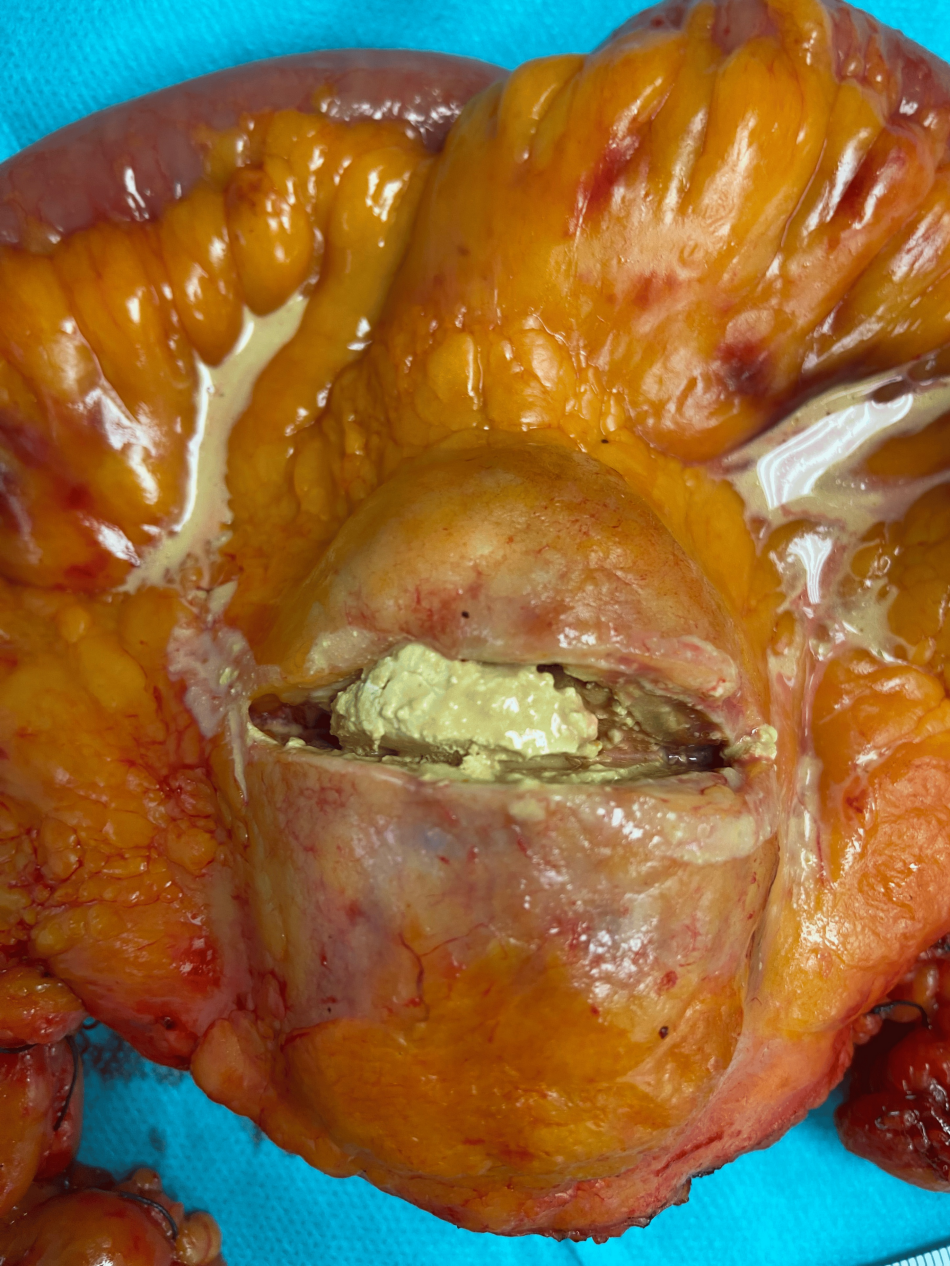
Opened mesentery mass showing central necrosis with a cheesy consistency.

**Figure 4:**
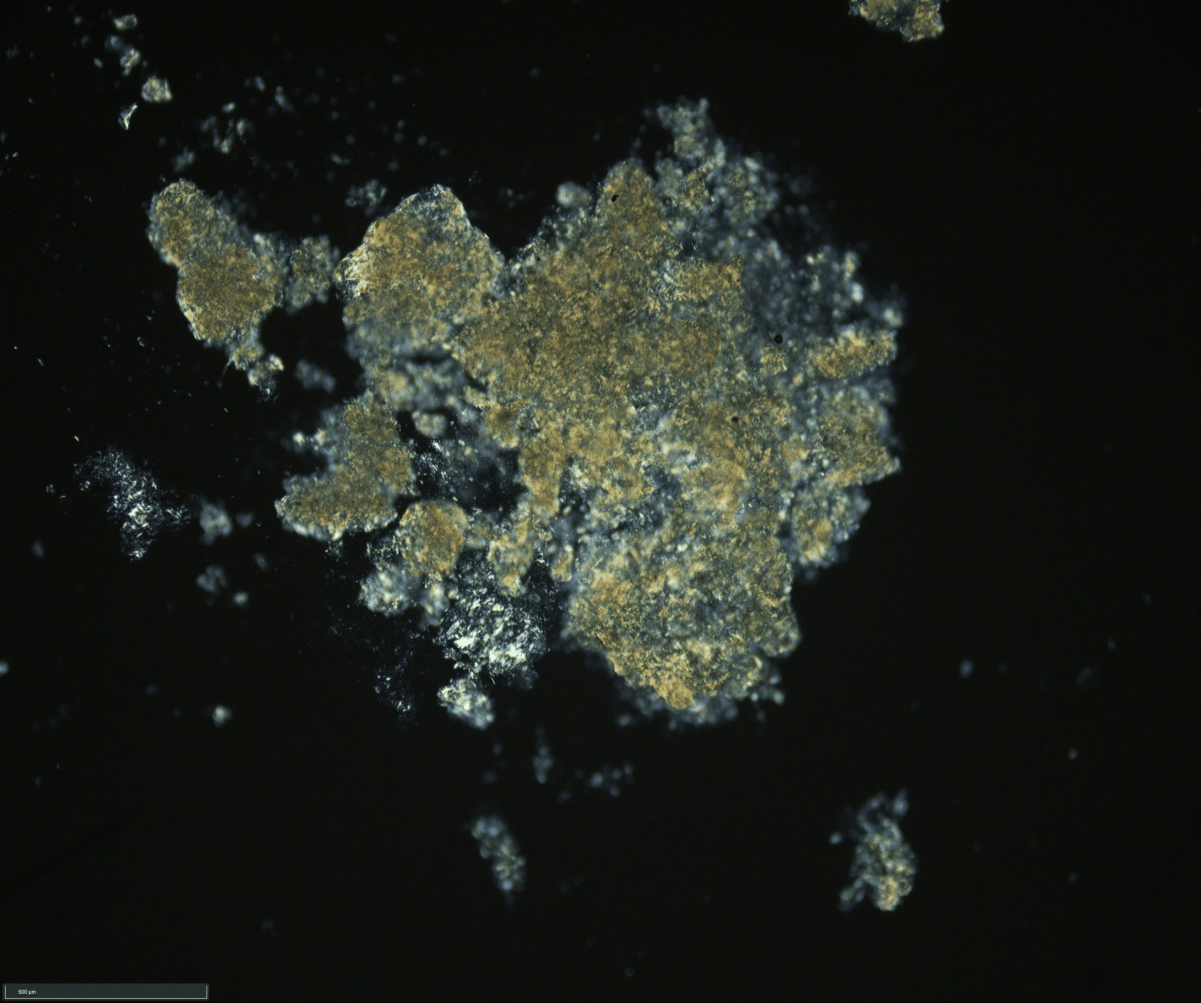
Native smear preparation from the intrapseudocapsular yellowish mass demonstrating optical birefringence compatible with gout crystals.

**Figure 5:**
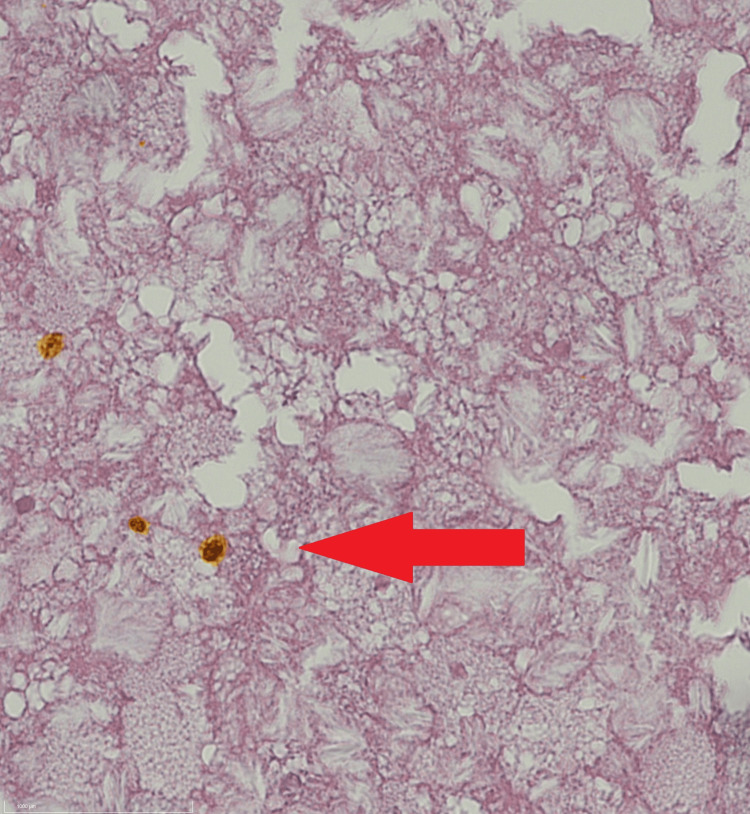
Paraffin-embedded tissue showing a thick pseudocapsule with ochre crystalloid precipitates and siderin pigmentation (arrow) (H&E, 100x magnification).

Macroscopic evaluation revealed a cystic mass with sharp borders, containing white to yellowish, doughy material (Figure 3). Smear preparations from the intrapseudocapsular material showed dark brown crystals, and examination under polarized light confirmed optical birefringence consistent with gout crystals (Figure 4). Histological sections showed a circumferential capsule-like boundary, varying in width, with mostly cell-poor areas and abundant hyaline-amorphous material. Clusters of yellow to dark brown crystalloid precipitates were observed, especially in the central region, with tuft-like crystalloid formations. Siderophages and a strong lymphatic infiltrate at the margins suggested residual lymph node tissue. The periphery of the lesion showed recurrent, brace-shaped dystrophic calcifications. Paraffin-embedded tissue revealed a thick pseudocapsule, with brown, yellow to ochre crystalloid precipitates and siderin pigmentation (Figure 5).

## Discussion

The detection of vague abdominal masses in radiological imaging presents diagnostic challenges. In this case, the patient’s symptoms were subacute, but many such masses are incidental findings or present as vague, long-standing digestive issues. CT scans are helpful in identifying mass location, contrast enhancement, fluid collections, or calcification. For further characterization, MRI imaging can provide better soft tissue contrast and assess tumor consistency and involvement with abdominal organs. In this case, MRI was not performed, as the CT scan clearly located the mass and showed no signs of organ or vessel invasion.

Differential diagnoses for calcifying fibrous tumors include GISTs, leiomyomas, schwannomas, solitary fibrous tumors, inflammatory myofibroblastic tumors, plexiform fibromyxomas, fibromatosis, sclerosing mesenteritis, and reactive nodular fibrous pseudotumors [15]. GISTs are the most commonly encountered lesions. However, in this case, the mass was determined to be a large gouty tophus, a rare manifestation of long-standing gout [16]. Recent studies suggest that systemic urate crystal deposition may be more clinically relevant than previously thought. There is evidence that urate crystals deposit in vessels, including coronary arteries, and contribute to the development of cardiovascular diseases, increasing the risk of sudden cardiac death [5]. Other uncommon locations for urate deposition include the spine, ocular structures, dermal tissue, renal parenchyma, and cardiac valves [17]. Intra-abdominal gout tophi have been described in a few reports, but this remains a rare condition [18,19]. One case involved an intra-abdominal, extra-intestinal pelvic mass mimicking an abscess, later identified as a gouty tophus.

## Conclusions

We report an extremely rare case of a gouty tophus in the mesentery of the small intestine. Despite the rarity of extra-articular gout manifestations, clinicians should consider the systemic effects of hyperuricemia in patients presenting with unexplained abdominal masses. Even in the absence of articular symptoms, urate crystal deposition in other tissues can present a risk factor for disease development.

## References

[1] Brook RA, Forsythe A, Smeeding JE, Lawrence Edwards N (2010). Chronic gout: epidemiology, disease progression, treatment and disease burden. Curr Med Res Opin.

[2] Riggs KR, Richman JS, Cherrington AL, Singh JA (2025). Allopurinol adherence in US patients with gout: analysis of the Medical Expenditure Panel Survey. J Clin Rheumatol.

[3] Te Kampe R, Janssen M, van Durme C, Jansen TL, Boonen A (2021). Sex differences in the clinical profile among patients with gout: cross-sectional analyses of an observational study. J Rheumatol.

[4] McClory J, Said N (2009). Gout in women. Med Health R I.

[5] Kleber ME, Delgado G, Grammer TB (2015). Uric acid and cardiovascular events: a Mendelian randomization study. J Am Soc Nephrol.

[6] Robinson PC (2018). Gout - an update of aetiology, genetics, co-morbidities and management. Maturitas.

[7] Mäki P, Harald K, Lindström J, Männistö S, Laatikainen T (2024). Association of adiposity with morbidity in Finnish adults: a register-based follow-up study. Scand J Public Health.

[8] Chhana A, Dalbeth N (2015). The gouty tophus: a review. Curr Rheumatol Rep.

[9] Eason A, House ME, Vincent Z (2016). Factors associated with change in radiographic damage scores in gout: a prospective observational study. Ann Rheum Dis.

[10] Ayoub I, Almaani S, Brodsky S, Nadasdy T, Prosek J, Hebert L, Rovin B (2016). Revisiting medullary tophi: a link between uric acid and progressive chronic kidney disease?. Clin Nephrol.

[11] Bajaj S, Fessler BJ, Alarcón GS (2004). Systemic lupus erythematosus and gouty arthritis: an uncommon association. Rheumatology (Oxford).

[12] Kiltz U, Alten R, Fleck M (2017). [Evidence-based recommendations for diagnostics and treatment of gouty arthritis in the specialist sector: S2e guidelines of the German Society of Rheumatology in cooperation with the AWMF]. Z Rheumatol.

[13] Shekelle PG, Newberry SJ, FitzGerald JD (2017). Management of gout: a systematic review in support of an American College of Physicians Clinical Practice Guideline. Ann Intern Med.

[14] Rider TG, Jordan KM (2010). The modern management of gout. Rheumatology (Oxford).

[15] Turbiville D, Zhang X (2020). Calcifying fibrous tumor of the gastrointestinal tract: a clinicopathologic review and update. World J Gastroenterol.

[16] Schottenfeld D, Beebe-Dimmer JL, Vigneau FD (2009). The epidemiology and pathogenesis of neoplasia in the small intestine. Ann Epidemiol.

[17] Härle P, Schlottmann K, Ehrenstein BP (2006). A patient with arthritis, severe back pain, impaired wound healing, and perforated sigmoid colon. Lancet.

[18] Wu H, Klein MJ, Stahl RE, Sanchez MA (2004). Intestinal pseudotumorous gouty nodulosis: a colonic tophus without manifestation of gouty arthritis. Hum Pathol.

[19] Chen CH, Chen CK, Yeh LR, Pan HB, Yang CF (2005). Intra-abdominal gout mimicking pelvic abscess. Skeletal Radiol.

